# 1,2-propanediol-trehalose mixture as a potent quantitative real-time PCR enhancer

**DOI:** 10.1186/1472-6750-11-41

**Published:** 2011-04-18

**Authors:** Helena Horáková, Iva Polakovičová, Gouse M Shaik, Jiří Eitler, Viktor Bugajev, Lubica Dráberová, Petr Dráber

**Affiliations:** 1 Department of Signal Transduction, Institute of Molecular Genetics, Academy of Sciences of the Czech Republic, Vídeňská 1083, 142 20 Prague 4, Czech Republic

## Abstract

**Background:**

Quantitative real-time PCR (qPCR) is becoming increasingly important for DNA genotyping and gene expression analysis. For continuous monitoring of the production of PCR amplicons DNA-intercalating dyes are widely used. Recently, we have introduced a new qPCR mix which showed improved amplification of medium-size genomic DNA fragments in the presence of DNA dye SYBR green I (SGI). In this study we tested whether the new PCR mix is also suitable for other DNA dyes used for qPCR and whether it can be applied for amplification of DNA fragments which are difficult to amplify.

**Results:**

We found that several DNA dyes (SGI, SYTO-9, SYTO-13, SYTO-82, EvaGreen, LCGreen or ResoLight) exhibited optimum qPCR performance in buffers of different salt composition. Fidelity assays demonstrated that the observed differences were not caused by changes in Taq DNA polymerase induced mutation frequencies in PCR mixes of different salt composition or containing different DNA dyes. In search for a PCR mix compatible with all the DNA dyes, and suitable for efficient amplification of difficult-to-amplify DNA templates, such as those in whole blood, of medium size and/or GC-rich, we found excellent performance of a PCR mix supplemented with 1 M 1,2-propanediol and 0.2 M trehalose (PT enhancer). These two additives together decreased DNA melting temperature and efficiently neutralized PCR inhibitors present in blood samples. They also made possible more efficient amplification of GC-rich templates than betaine and other previously described additives. Furthermore, amplification in the presence of PT enhancer increased the robustness and performance of routinely used qPCRs with short amplicons.

**Conclusions:**

The combined data indicate that PCR mixes supplemented with PT enhancer are suitable for DNA amplification in the presence of various DNA dyes and for a variety of templates which otherwise can be amplified with difficulty.

## Background

Advances in the methodology of qPCR contributed significantly to a widespread use of this method for DNA genotyping, gene expression analysis and mutational scanning. Several different systems have been developed for continuous monitoring of the production of PCR amplicons and characterization of their properties. Widely used are sequence-specific probes which facilitate a highly sensitive detection of specific PCR products. However, these probes are difficult to prepare and are relatively expensive [1]. An alternative to the probe-based methods is the use of DNA-intercalating dyes which at concentrations compatible with PCR-mediated DNA amplification exhibit enhanced fluorescence after binding to double-stranded (ds)DNA. These dyes are less expensive, but they are also less specific because they bind to all dsDNAs present in PCR mixtures, including nonspecific products and primer-dimers. Although some of these unwanted DNA species can be distinguished by analysis of the melting curves of PCR amplicons, their presence reduces the sensitivity of qPCR and requires a proper adjustment of PCR conditions. Biophysical studies showed that DNA dyes bind to dsDNA by intercalation and external binding, and that these interactions could interfere with PCR [2-4]. Furthermore, it has been shown that the dyes also react with single-stranded (ss)DNA oligonucleotide primers [2] and that this binding could inhibit annealing of the primers to the template during PCR [5]. This could account for some difficulties in amplifying certain DNA fragments, which are otherwise easily amplified in the absence of the dyes.

In initial studies, real-time accumulation of PCR amplicons was evaluated with ethidium bromide [6]. This dye was later substituted with SGI [7], which quickly became the most-widely used DNA dye for qPCR monitoring. Recently, several other DNA dyes have been introduced giving a strong fluorescence signal with dsDNA at concentrations not inhibiting PCR. These include YO-PRO-1 [8], BEBO [9], LCGreen [10], SYTO-9 [4,11], EvaGreen [3], SYTO-13, SYTO-82 [11] and LightCycler 480 ResoLight dye [12,13].

We have found that SGI inhibits amplification of medium-size genomic DNA fragments and that this inhibitory effect can be reduced by using a PCR mix, denoted here as mix IV, with modified salt composition [5]. In this study, we compared qPCR performance of seven DNA dyes (Table 1) in the mix IV and three other widely used PCR mixes of different salt composition. We found that amplification in the presence of SGI was optimal in mix IV, whereas all other dyes performed better in a mix marked here as mix II. To find out conditions which would allow efficient amplification of difficult-to-amplify DNA templates, such as those in whole blood and/or GC-rich and compatible with various DNA dyes, we tested various additives and their combinations. Excellent performance was found when PCR mix II was supplemented with PT enhancer. Extensive testing showed that PT enhancer-containing mix II could be used for efficient amplification of various DNA templates known to resist amplification under various routinely used conditions. The data have implications for a more rational design and routine use of qPCR assays.

**Table 1:** DNA dyes, their origin and properties

DNA dye	Origin	Stock concentration	Final concentration	Absorption maximum	Emission maximum
SGI	Invitrogen	10 mM in DMSO*	0.33 μM	497	520
SYTO-9	Invitrogen	5 mM in DMSO	2 μM	485	498
SYTO-13	Invitrogen	5 mM in DMSO	2 μM	488	509
SYTO-82	Invitrogen	5 mM in DMSO	2 μM	541	560
EvaGreen	Biotium	25 mM in DMSO	1.33 μM	500	530
LCGreen	Idaho	10 × concentrated	1×	440-470	470-520
ResoLight	Roche	20 × concentrated	1×	450-500	487

* [3]

## Results

### PCR with difficult-to-amplify templates

**Figure 1:**
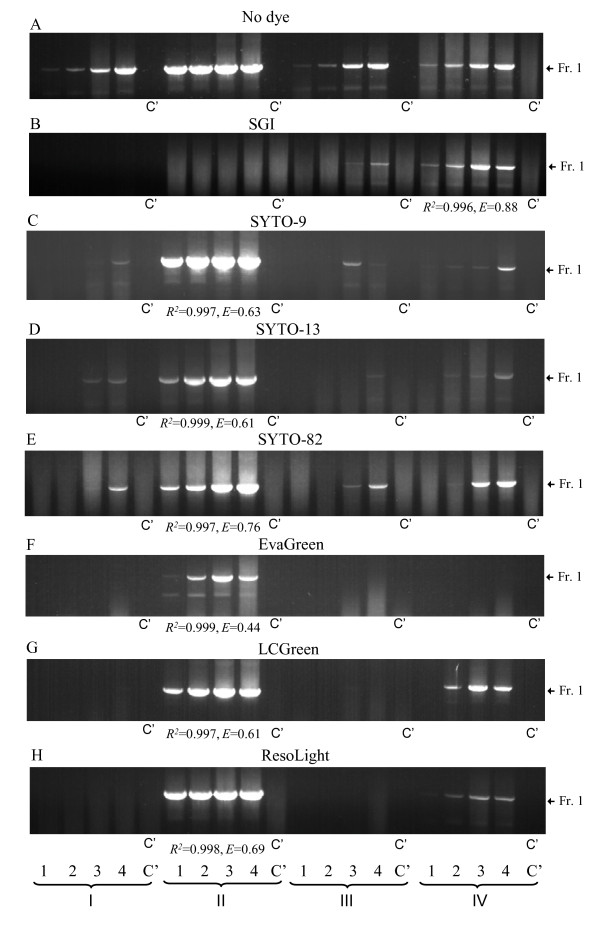
**Amplification of genomic DNA fragments in various combinations of PCR mixes and DNA dyes**. Isolated genomic DNA was diluted to contain 47 (1), 141 (2), 423 (3) 1269 (4) or none (Control; C') copies of Thy-1 gene per one μl of different PCR mixes (I - IV) supplemented without (A) or with (B - H) various DNA-binding dyes. The samples were analyzed by PCR (A) or qPCR (B - H) followed by agarose gel electrophoresis; DNA fragments were visualized by ethidium bromide staining. Migration of the specific DNA fragment (864 bp) is indicated by an arrow (Fr. 1). Regression coefficients (*R*^*2*^) and efficiencies (*E*) were calculated from plots of Cq values versus log copy numbers, and are presented only for samples with the expected melting temperature at all template dilutions. All samples were analyzed in 3 - 7 independent experiments with similar results.

In our previous study we showed that amplification of the 864 base pairs (bp) genomic fragment of mouse Thy-1 can be achieved only in a PCR mix denoted here as mix IV [5]. In this study, we first tested whether the mix IV was also optimal for qPCR analysis with other DNA dyes. We compared amplification of Thy-1 genomic DNA fragment in mix IV and in three other widely used PCR mixes combined with seven DNA dyes. Properties of all DNA dyes and composition of all PCR mixes used are shown in Table 1 and 2, respectively. When SGI was combined with PCR mix IV, amplification was observed at all dilutions of the template DNA with reasonable regression coefficient (*R*^2 ^= 0.996 ± 0.003; mean ± SD; n = 6) and efficiency (*E *= 0.88 ± 0.11). In all other mixes amplification of the fragment was either absent (mix I and II) or poor (mix III). These data were confirmed by agarose gel electrophoresis (Figure 1B). Poor amplification in PCR mixes I - III was obviously caused by the presence of SGI, since Thy-1 was reproducibly amplified in its absence (Figure 1A). Changing the Mg^2+ ^concentration or optimizing the annealing temperature failed to improve the reaction in mixes I - III. It should be noted that the amount of Thy-1 amplicons in mix IV was similar regardless of the presence or absence of SGI (compare Figure 1A and 1B), indicating that the salt composition of PCR mixes has a decisive role on the inhibitory effect of SGI.

**Table 2:** PCR mixes used and their composition

	PCR mixes*			
	
Component**	I	II	III	IV
Tris-HCl (mM) [pH]***	10 [8.0]	75 [8.8]	10 [ 8.0]	20 [8.8]
KCl (mM)	50	-	50	10
(NH_4_)_2_SO_4 _(mM)	-	20	-	10
MgSO_4 _(mM)	-	-	-	2
Triton X-100 (%)	0.1	-	0.1	0.1
Tween 20 (%)	-	0.01	-	-
MgCl_2 _mM	2.5	2.5	2.5	-
DMSO (%)	-	-	5	-
dNTPs μM	200	200	200	200
Taq DNA pol. (U/ml)	25	25	25	25
anti-Taq mAb (nM)	22	22	22	22

* Final concentrations of the component during PCR.

** Components present at the same concentrations in all mixes include 200 μM dNTPs, Taq DNA pol. (25 U/ml) and anti-Taq mAb (22 nM). Oligonucleotide primers, DNA templates, DNA dyes and additives/enhancers were added immediately before the assay.

*** pH at 21°C.

When the same fragment was amplified in SYTO-9-supplemented PCR mixes, a different profile was obtained: strong amplification (*R*^2 ^= 0.997, *E *= 0.63) was observed in mix II, but only week and variable amplification, as detected by melting curve analysis and gel electrophoresis, was observed in mixes I, III and IV (Figure 1C). Mix II was also optimal for other DNA dyes (SYTO-13, SYTO-82, EvaGreen, LCGreen and ResoLight; Figure 1D - H). Interestingly, inhibition of PCRs by different dyes varied in different mixes. In mix I, for example, amplification of Thy-1 DNA fragment was only observed at high concentrations of the template in the following order: SYTO-82 > SYTO-9 = SYTO-13. In mix III, some reactivity was observed with the following dyes: SYTO-82 > SGI = SYTO-9 = SYTO-13. In mix IV, Thy-1 amplification was seen in mixes supplemented with SGI > LCGreen > SYTO-82 > ResoLight > SYTO-9 = SYTO-13. In several combinations of PCR mixes and DNA dyes, complete inhibition of amplification was noted: SGI in mixes I and II, EvaGreen in mixes I, III and IV and LCGreen and ResoLight in mixes I and III. This inhibition persisted even when concentration of MgCl_2 _was raised up to 5 mM (data not shown).

### Fidelity assays

The observed difficulties with amplification of medium-size genomic DNA fragments in some PCR mixes might in part reflect an enhanced mutagenesis interfering with the synthesis of DNA fragments [14]. To find out whether salt composition of PCR mixes and the presence of DNA dyes could affect the fidelity of PCRs, we used an assay system based on streptomycin resistance of *rps*L mutants [15]. Data presented in Table 3 show that in mix I the error rate was 3.88 × 10^-7^. A similar error rate was observed in mix II and an approximately two-fold increase in mix III and IV. To prove the sensitivity of the fidelity assay, we also amplified the template using KOD polymerase in a reaction mix of undisclosed composition provided by the manufacturer of the enzyme. As expected, amplification with KOD polymerase resulted in substantially lower error rate (7.6 × 10^-5^), which is in agreement with the manufacturer's data. When PCR mix II was supplemented with SYTO-9, SYTO-13, EvaGreen, LCGreen or ResoLight, no dramatic changes in mutation frequencies were observed (Table 3). These findings indicate that different mutation frequencies in different PCR mixes cannot by themselves explain the observed changes in amplification efficiency caused by various DNA dyes.

**Table 3:** Effect of the tested PCR mixes and selected DNA dyes on DNA polymerase fidelity

Sample	Colonies mutant/total	**Template doubling**^**a**^	**Mutation frequency**^**b**^	**Error rate (× 10**^**-6**^**)**^**c**^
*Taq *in mix I	362/5789	12.5	0.063	38.8
*Taq *in mix II	990/16342	10.9	0.061	43.0
*Taq *in mix III	2760/21433	12.4	0.129	80.0
*Taq *in mix IV	951/8273	11.7	0.115	75.6
KOD in mix K^d^	30/25006	12.1	0.0012	0.76

Various DNA dyes in PCR mix II				
SYTO-9	318/4845	9.1	0.066	46.0
SYTO-13	640/10087	9.2	0.063	44.7
EvaGreen	307/5607	9.2	0.054	38.6
LCGreen	211/4068	9.0	0.052	35.8
ResoLight	64/1243	9.1	0.052	36.2

^a ^Template doublings (d) were calculated using the equation 2^d ^= (amount of PCR product)/(amount of starting target).

^b ^Mutation frequency = (mutant colonies)/(total colonies).

^c ^Error rate = (mutation frequency)/(130 × d).

^d ^PCR mix provided by KOD enzyme manufacturer

### New universal PCR master mix

**Figure 2:**
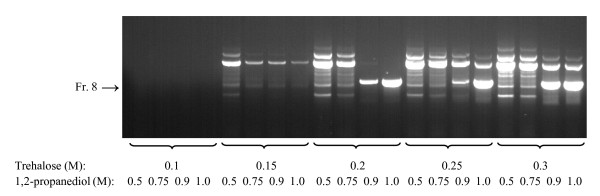
**Amplification of medium-size GC-rich DNA fragment using whole blood as a source of the template**. 806 bp fragment of human Q8N1R6 gene (73.3% GC) in heparinized human blood (2% final concentration) was amplified in PCR mix II containing various concentrations of trehalose (0.1 - 0.3 M) and 1,2-propanediol (0.5 - 1.0 M). Position of the specific product (Fr. 8) is shown by an arrow. A typical result of three independently performed experiments is shown.

In an attempt to develop a universal PCR master mix compatible with all the DNA dyes and suitable for amplification of DNA templates that cannot be readily amplified due to dye interference, presence of inhibitory substances and/or secondary structure formation, we tested several additives combined with mixes I - IV and various DNA dyes. As a template we used GC-rich DNA fragment of Q8N1R6 gene (Table 4; 806 bp, 73.3% GC) in human heparinized blood which escaped detection under standard conditions using various commercial PCR master mixes such as iQ™ SYBR Green Supermix and LightCycler 480 SYBR Green I Master (LC 480 SGI). In pilot experiments various mixes were combined with several additives and/or procedures, which have been reported to allow amplification of GC-rich fragments and/or neutralize PCR inhibitory components present in the blood (hemoglobin, lactoferrin and immunoglobulin G [16,17]). These included 0.1 - 0.5 M trehalose (final concentration) [18], 5 - 15% dimethyl sulfoxide (DMSO) [19,20], 0.5 - 2.5 M N,N,N-trimethylglycine monohydrate (betaine) [21,22], combinations of 5 - 15% DMSO and 2.2 M betaine [23], 5 - 25 mM tetrapropylammonium chloride [5], 0.5 - 1.5 M 1,2-propanediol, 0.5 - 1.5 M ethyleneglycol [24], 50 - 150 μM 7-deaza-2'-deoxyguanosine 5'-triphosphate [25,26], PCR-enhancing coctail containing 0.3 M D-(+)-trehalose, 0.24 M L-carnitine, and 0.4% Nonidet P-40 (TCN) [27] and antibody-mediated hot start PCR [5] combined with "touchdown" procedure [28,29]. Yet, none of these additives and/or procedures improved PCR to get specific signal determined by agarose gel electrophoresis (data not shown). Interestingly, specific amplicons were observed in PCR mix II supplemented with both 1 M 1,2-propanediol and 0.2 M trehalose (mix II-PT); higher or lower concentrations of these two additives resulted in inhibition of production of the specific amplicons and/or enhanced formation of nonspecific DNA fragments (Figure 2).

**Table 4:** Oligonucleotide primer sets and PCR amplicon properties

No	Gene*	Chromosome		Amplicon		Primer (5'-3')
				
	Name	**No**.	Position (Start-End)**	%GC	Fragment (bp)/name	Forward/reverse
Pr. 1	Thy-1	9	43854065-43854933	53.6	864/Fr. 1	ATGAACCCAGCCATCAGCG/GGGTAAGGACCTTGATATAGG
						
Pr. 2	NTAL	5	135085842-135086225	50.8	384/Fr. 2	CTAGGGAGCTGAGTGTTCTCA/GAACGGCTAGAACTACACAGAG
						
Pr. 3	NP_660313.1	16	613645-614375	71.5	731/Fr. 3	GGTCGCCGACATCCACTC/TGCTCCGGGAACAGAACCT
						
Pr. 4	Q8WZ58	11	2292033-2292813	71.7	781/Fr. 4	CCTGACCGTCCTGGCACA/CTGGCGAAATCTGCGAGTTC
						
Pr. 5	NP_060193.2	9	140174905-140175662	72.4	758/Fr. 5	CCCCTCACTCAGGTCGTGTTTT/CCCTCTGAGCCCCTTTCG
						
Pr. 6	Q8N4X1	16	2029023-2029774	72.6	752/Fr. 6	CGGTCCATCCCCTCATCG/ACCCCTCACGCCACCAC
						
Pr. 7	NP_001035158.1	16	33961629-33962363	73.1	735/Fr. 7	CGGCGAACCGGACATCC/GGCTCGTGAGGCGGGTCT
						
Pr. 8	Q8N1R6	13	111267817-111268622	73.3	806/Fr. 8	GACACGGCCCTGCTCC/GGGTGTGATTGAGCGAGTTG
						
Pr. 9	NM_020975	10	43572333-43572724	79.1	392/Fr. 9	CCCGCACTGAGCTCCTACAC/GGACGTCGCCTTCGCCATCG

* Homo_sapiens LATESTGP database was used, except for Thy-1 and NTAL for which Mus_musculus LATESTGP database was used.

** Primer sets Pr. 3 - Pr. 9. are based on Ensembl release 56 - Sept 2009

**Figure 3:**
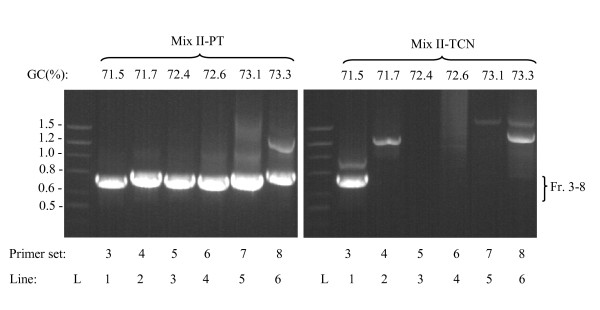
**Amplification of various GC-rich genomic DNA fragments from whole human blood**. PCR mix II was supplemented with 1 M 1,2-propanediol and 0.2 M trehalose (final concentrations; Mix II-PT) or 0.3 M trehalose, 0.24 M L-carnitine, and 0.4% Nonidet P-40 (Mix II-TCN). Various GC-rich templates from heparinized whole human blood (~100 copies of the template/μl of PCR mixture) were amplified in the two PCR mixes using primer sets specified in Table 4. Positions of the specific fragments (731-806 bp in size) are indicated on the right (Fr. 3-8). Lane L, DNA standard ladder with sizes indicated in kbp on the left. The numbers at the top indicate percentages of GCs in specific amplicons. A typical experiment of at least four performed is shown.

Mix II-PT allowed efficient amplification of numerous gene fragments in heparin-treated whole blood, including mouse Thy-1 and NTAL (data not shown) and, as shown in Figure 3 (mix II-PT), GC-rich fragments of the human genes NP_660313.1 (Table 4; 71.5% GC, 731 bp), Q8WZ58 (71.7% GC, 781 bp), NP_060193.2 (72.4% GC, 758 bp), Q8N4X1 (72.6% GC, 752 bp), NP_001035158.1 (73.1% GC, 735 bp), and Q8N1R6 (73.3% GC, 806 bp). PT enhancer also allowed efficient amplification of 392 bp DNA fragment with 79.1% GC (Table 4; [26]) from whole blood which was resistant to amplification under standard conditions (data not shown). In its enhancing capacity mix II-PT surpassed the TCN enhancer [27], which was capable of amplifying only one of the six GC-rich templates tested (Figure 3, mix II-TCN). Similar results were obtained when blood samples were treated with other commonly used anticoagulants, 2.7 mM EDTA or 0.38% sodium citrate (data not shown).

**Figure 4:**
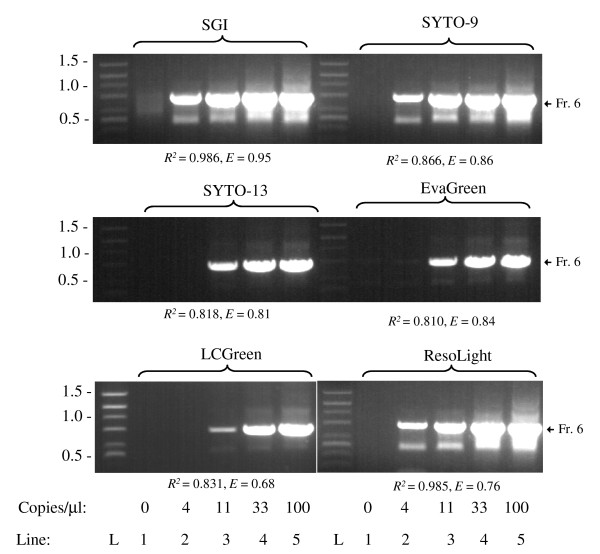
**Amplification of GC-rich DNA fragment from whole blood in the presence of various DNA dyes**. Human blood was diluted with mouse blood to yield 0, 4, 11, 33 and 100 copies of human Q8N4X1 gene per 1 μl of PCR reaction mix II supplemented with PT enhancer and various DNA dyes. Final concentration of human/mouse blood was 2% in all mixes. The samples were analyzed by qPCR followed by agarose gel electrophoresis and staining with ethidium bromide. Position of the specifically amplified DNA fragment is indicated by an arrow (Fr. 6). Lane L, DNA standard ladder with sizes indicated in kbp on the left. Regression coefficients (*R*^*2*^) and efficiencies (*E*) were calculated from plots of Cq values versus log copy numbers. All samples were analyzed in 3-4 independent experiments with similar results.

Next we tested qPCR performance of various DNA dyes in mix II-PT during amplification of the GC-rich fragment (72.6% GC, 752 bp) of Q8N4X1 gene in whole human blood. Pilot experiments indicated that different dilutions of whole human blood in water, followed by qPCR in mix II-PT with SGI resulted in poor regression coefficients. This was probably caused by simultaneous dilution of the DNA templates and the inhibitory (e.g. hemoglobin [17]) and/or stimulatory (e.g. heparin [30]) components present in the whole blood samples. However, when human blood was diluted with mouse blood to keep the concentration of human/mouse blood in PCR constant (2%), reasonable regression coefficients (*R*^*2 *^= 0.986 ± 0.006; mean ± S.D.; n = 4) and efficiencies (*E *= 0.95 ± 0.03) were obtained. Amplification of nonspecific fragments after 40 cycles of PCR was low, as determined by agarose gel electrophoresis (Figure 4, top, left). Under the same conditions other DNA dyes also gave satisfactory results in the following order (based on regression coefficients): SGI > ResoLight > SYTO-9 > LCGreen > SYTO-13 > EvaGreen (Figure 4). Thus, Mix II-PT is unique in its capability to serve as universal qPCR mix for all DNA dyes tested.

**Figure 5:**
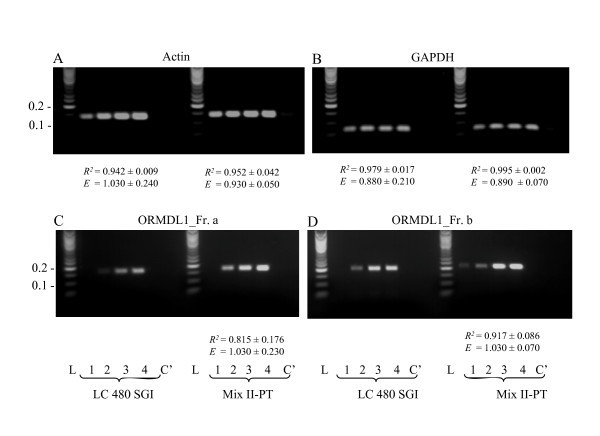
**Enhanced performance of PCR mix II-PT in routine qPCR amplifications**. cDNA from mouse bone marrow-derived mast cells was prepared, diluted 1:10^5 ^(1), 1:10^4 ^(2), 1:10^3 ^(3) or 1:10^2 ^(4) and amplified in SGI-supplemented PCR mix II-PT or LC 480 SGI using primer sets for Actin, GAPDH, ORMDL1_Fr. a or ORMDL1_Fr. b. In controls (C'), cDNA was replaced by H_2_O. The samples were analyzed by qPCR followed by agarose gel electrophoresis and staining with ethidium bromide. Lane L, DNA standard ladder with sizes indicated in kbp on the left. Regression coefficients (*R*^*2*^) and efficiencies (*E*) were calculated from the plots of Cq values versus log copy numbers and are presented only for samples with the expected melting temperature at all template dilutions. Data are presented as means ± S.D. calculated from three independent experiments performed in triplicates.

PCR mix II-PT could be used with advantage not only for amplification of DNA fragments from crude blood samples that are difficult to amplify, but also for routine qPCR analysis of cDNA fragments without time-consuming adjustment of qPCR conditions for individual primer sets. Data presented in Figure 5A show that for amplification of 138 bp fragment of actin cDNA (58% GC) mix II-PT supplemented with SGI gave comparable regression coefficients and efficiencies as the routinely used LC 480 SGI. Similar results were obtained when GAPDH cDNA fragment was analyzed (Figure 5B; 52% GC, 69 bp). However, when low abundant cDNA fragments were amplified, such as ORMDL1_Fr.a (Figure 5C; 43% GC, 171 bp) or ORMDL1_Fr.b (Figure 5D; 47% GC, 205 bp), detectable amplification at all concentrations of template cDNA and reasonable regression coefficients and efficiencies were obtained only in mix II-PT-supplemented samples. Interestingly, addition of 1,2-propanediol and/or trehalose at various concentrations to LC 480 SGI containing chemically modified Taq DNA polymerase did not improve performance of this PCR mix, but instead had an inhibitory effect (data not shown).

### Fluorescence measurements and melting temperatures

**Figure 6:**
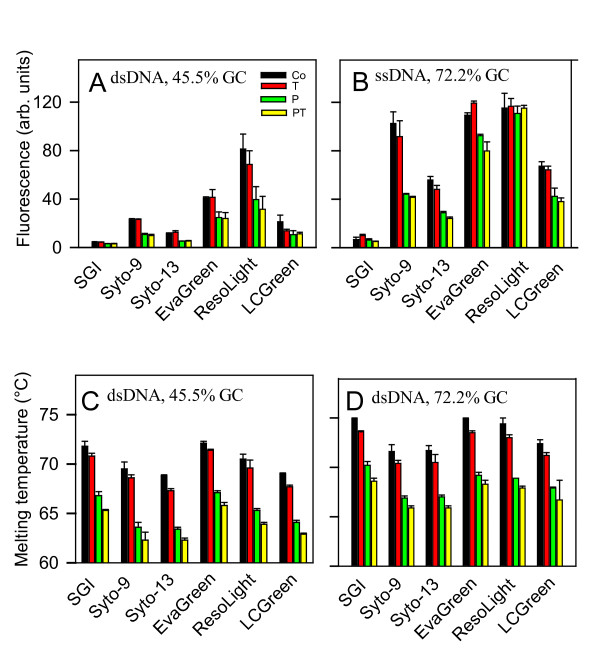
**The effect of various DNA dyes and enhancers on ssDNA fluorescence and dsDNA melting temperature**. (A) TNF-1 oligonucleotide (ssDNA, 45.5% GC; 1 μM final concentration) in PCR mix II (without dNTPs, Taq DNA polymerase and anti-Taq) was mixed with H_2_O (Control; Co) or enhancers [0.2 M trehalose (T; final concentration), 1 M 1,2-propanediol (P) or both 1 M 1,2-propanediol and 0.2 M trehalose (PT)] and various DNA dyes at final concentrations as indicated in Table 1. After heating at 95°C for 2 min the samples were cooled to 50°C and fluorescence was determined using Mastercycler ep realplex. (B) Oligonucleotide primer No 7, reverse (ssDNA; 72.2% GC; 1 μM final concentration) in PCR mix II was combined with various additives and DNA dyes, and fluorescence at 50°C was determined as in A. (C) Oligonucleotide mixture of TNF-1 and anti-TNF-1 (dsDNA, 45.5% GC; 1 μM final concentration) was prepared in mix II supplemented with various additives and DNA dyes. The samples were heated to 95°C for 2 min, then cooled to 30°C and temperature-dependent changes in fluorescence were obtained during heating from 30 to 95°C (0.2°C increments) in Mastercycler ep realplex. Melting temperatures were determined from the melting curves. (D) Oligonucleotide mixture of the primer No 7, reverse, and the anti-primer 7 (dsDNA, 72.2% GC; final concentration 1 μM) was combined in mix II with additives and DNA dyes and analyzed as in C. Means ± S.D. were calculated from 3 - 5 measurements.

In our previous study we showed that SGI bound to ssDNA primers and suggested that the binding could at least in part contribute to the inhibitory effect of SGI in qPCR assays [5]. In further studies we evaluated whether trehalose and/or 1,2-propanediol could interfere with interaction of various DNA dyes with ssDNA, as reflected by changes in fluorescence signal. Data presented in Figure 6A indicate that interaction of various DNA dyes (at concentrations used for qPCR) with ssDNA oligonucleotide primer for tumor necrosis factor (TNF; 45.5% GC) induced fluorescence in the following order: SGI < SYTO-13 < LCGreen < SYTO-9 < EvaGreen < ResoLight. Addition of 0.2 M trehalose had little to no inhibitory effect on this fluorescence. In contrast, 1,2-propanediol significantly (P < 0.05; n = 3-5) decreased fluorescence in all DNA dyes used, except for SGI. Combination of both 1,2-propanediol and trehalose had a similar effect as 1,2-propanediol alone. When ssDNA primer No. 7, reverse (Table 4; 72.2% GC) was used, basal level of fluorescence was increased in all DNA dye-enhancer combinations and again trehalose had no significant effect on fluorescence intensity. In contrast to trehalose, 1,2-propanediol significantly (P < 0.05; n = 4) decreased fluorescence, except for SGI and ResoLight (Figure 6B).

In an attempt to understand the enhancing effect of trehalose and 1,2-propanediol on qPCR performance, we also evaluated the melting temperatures of short dsDNA oligonucleotides. When dsDNA of TNF and anti-TNF (45.5% GC) was used, the melting temperature depended on DNA dye used (Figure 6C). The highest melting temperature was observed in control mix II supplemented with EvaGreen, followed by SGI > ResoLight > Syto-9 > LCGreen > Syto-13. Addition of 0.2 M trehalose decreased the melting temperature by 0.7 - 1.5°C, whereas addition of 1 M 1,2-propanediol decreased it by 4.9 - 5.9°C. When both trehalose and 1,2-propanediol were used, further decrease by 1.1 - 1.5°C was observed. Using dsDNA oligonucleotide with 72.2% GC, melting temperature was increased but a similar effect of trehalose and 1,2-propanediol was observed (Figure 6D).

### Cooperative effect of 1,2-propanediol and trehalose

**Figure 7:**
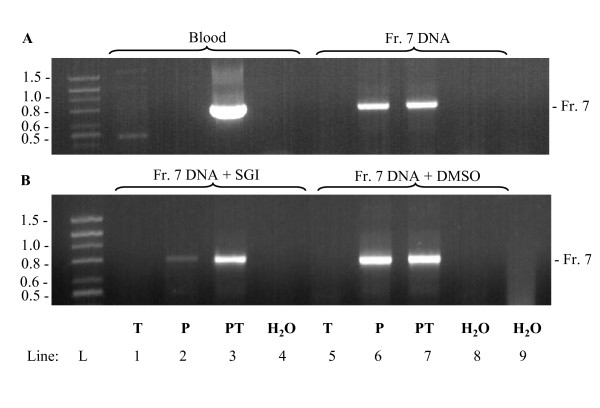
**Cooperative effect of 1,2-propanediol and trehalose on amplification of difficult-to-amplify DNA templates**. (A) DNA fragment of the human gene NP_001035158.1 (Fr. 7; 735 bp; 73.1% GC) was amplified from the whole blood (2% final) in PCR mix II supplemented with 0.2 M trehalose (T, final concentration), 1M 1,2-propanediol (P) or mixture of both (PT). Alternatively, PCR-amplified DNA (Fr. 7) was diluted in H_2_O and used as a template for PCRs performed under various conditions as above. PCR mixes supplemented with H_2_O instead of enhancers (line 4 and 8) and a mix without enhancers and template (line 9) served as controls. After amplification the fragments were analyzed by agarose gel electrophoresis and stained with ethidium bromide. (B) PCR amplified DNA (Fr. 7) was re-amplified and analyzed as in A, except that PCR mix II was supplemented with SGI (left) or vehicle (DMSO) alone (right). After amplification the fragments were analyzed by agarose gel electrophoresis and stained with ethidium bromide. Numbers to the left indicate migration of DNA ladder (L; in kbp). Gels from a typical experiment of at least four performed are shown.

The above data suggested that 1,2-propanediol and trehalose have different roles in promoting DNA amplification of GC-rich DNA fragments from crude blood samples: trehalose mainly acts by neutralizing the inhibitory components present in blood, whereas 1,2-propanediol mainly acts by decreasing melting temperature. To prove this, we compared the effect of the enhancers on amplification of GC-rich fragments from whole blood or isolated DNA. As expected, efficient amplification of DNA template in whole blood, occurred only in samples supplemented with both 1,2-propanediol and trehalose (Figure 7A, line 3). In contrast, when isolated GC-rich DNA fragment was used as a template, comparable amplification was observed in samples supplemented with 1,2-propanediol alone or together with trehalose (Figure 7A, lines 6 and 7). Trehalose alone was not able to promote amplification of the DNA templates (Figure 7A, lines 1 and 5).

When PCR mix was supplemented with SGI and isolated DNA was amplified, a different picture was observed. SGI partially inhibited amplification in 1,2-propanediol-supplemented samples (Figure 7B, line 2). This inhibition was caused by SGI as indicated by no inhibition in samples containing vehicle (DMSO) instead of SGI (Figure 7B, line 6). PCR mixes supplemented with both 1,2-propanediol and trehalose exhibited an improved amplification in SGI-supplemented samples (Figure 7B, line 3) indicating cooperative effect of the enhancers in SGI-supplemented samples.

**Figure 8:**
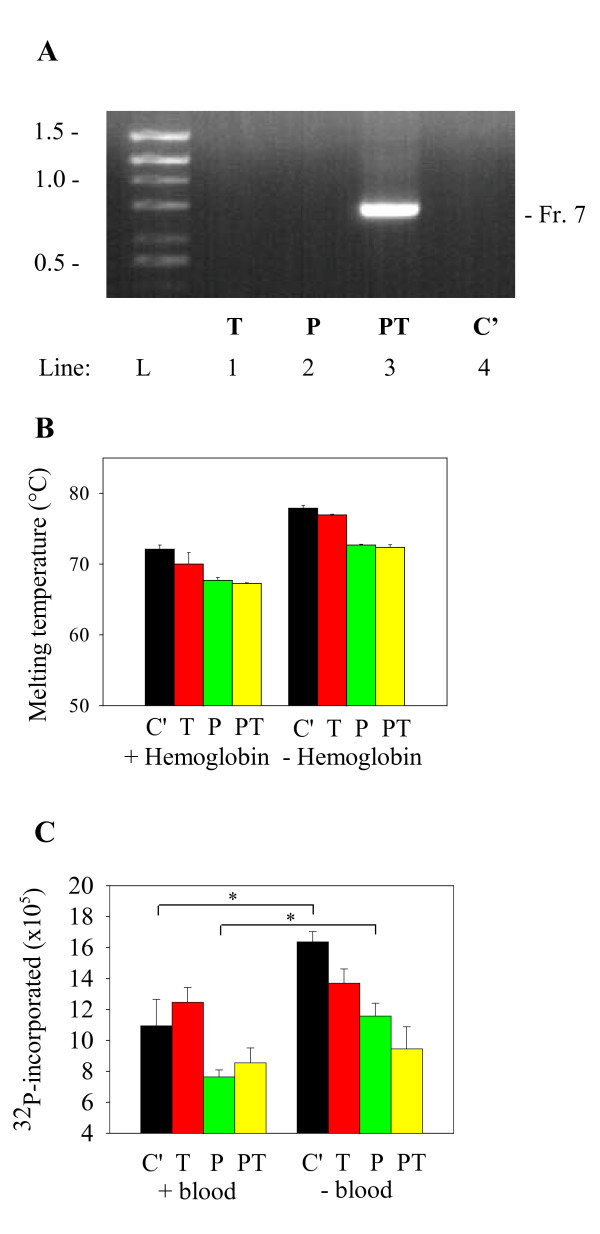
**The effect of blood inhibitors and enhancers on PCR, melting temperature, and polymerase activity**. (A) DNA fragment of NP_001035158.1 gene (Fr. 7; 735 bp; 73.1% GC) was amplified in PCR mix II supplemented with the corresponding primers, 37.5 μM hemoglobin and 0.2 M trehalose alone (T), 1 M 1,2-propanediol alone (P) or their combination (PT). PCR mix supplemented with H_2_O instead of enhancers served as a control (C'). A typical experiment of four performed is shown. (B) Melting temperature of GC-rich dsDNA oligonucleotide primer No 7, reverse, and the anti-primer (72.2% GC; 1 μM final concentration) in the presence (+) or absence (-) of hemoglobin (37.5 μM) and various enhancers (as in A). Melting temperature was determined as in Figure 6, except that SGI was used at higher concentration (1.32 μM). (C) Enzymatic activity of Taq DNA polymerase in PCR mix II buffer supplemented with (+) or without (-) 10% blood and various enhancers (as in A). Samples supplemented with H_2_O instead of enhancers (C') served as controls. Data in B and C indicate means ± S.D. (n = 4). Asterisks indicate statistically significant differences (P < 0.05).

Next we tested the effect of trehalose and 1,2-propanediol on amplification of isolated DNA in samples supplemented with hemoglobin. Pilot experiments showed that hemoglobin completely inhibited PCR at concentrations 25 μM and higher (data not shown). Data in Figure 8A indicate that the inhibitory effect of hemoglobin (37.5 μM) was removed by addition 1,2-propanediol and trehalose together but not by trehalose or 1,2-propanediol alone.

To understand the effect of the enhancers on amplification in the presence of blood inhibitors, we analyzed melting temperatures of dsDNA. We found that hemoglobin at the inhibitory concentration (37.5 μM) significantly decreased melting temperature of short dsDNA fragments by 5.8 ± 0.6°C (mean ± S.D.; n = 4). Addition of trehalose, 1,2-propanediol or both enhancers to hemoglobin-supplemented samples further decreased Tm by 2.1 ± 1.6°C, 4.4 ± 0.6°C and 4.8 ± 0.6°C, respectively (Figure 8B). These data suggested direct or indirect interaction of hemoglobin with DNA. The direct interaction was however weak, if any, as indicated by similar mobility in agarose gel of DNA fragments alone and DNA fragments mixed with hemoglobin (37.5 μM; data not shown).

Finally, we tested whether blood inhibitors could interfere with enzymatic activity of Taq DNA polymerase as determined by incorporation of [α-^32^P]dATP into activated salmon testes DNA. Data presented in Figure 8C indicate that blood at the inhibitory concentration (10%) significantly reduced activity of Taq DNA polymerase. Addition of trehalose significantly (P < 0.05) decreased the enzymatic activity of Taq DNA polymerase in control samples (- blood), but slightly increased the activity in blood-supplemented samples, leading to statistically non-significant differences between the control and blood-supplemented samples. 1,2-propanediol decreased enzymatic activity of Taq DNA polymerase in both control and blood-supplemented samples; the difference between these two groups was statistically significant. Addition of trehalose to 1,2-propanediol-supplemented control samples (PT) further significantly decreased (P < 0.05) the activity of Taq polymerase. In contrast, in blood-supplemented samples there was small (insignificant) increase in activity of Taq DNA polymerase in PT-containing samples compared to samples containing 1,2-propanediol alone. This led to non-significant differences between control and blood-containing samples with PT. These data indicate that trehalose has a different effect on activity of Taq polymerase in control and blood containing samples. This could explain at least in part the enhancing effect of trehalose in samples containing blood inhibitors.

## Discussion

The aim of this study was to develop new qPCR mixes capable of amplifying difficult DNA templates, such as those in whole blood, of medium size and/or GC-rich, in the presence of various DNA dyes. First, we assessed the properties of seven different DNA dyes in four widely used PCR buffers differing in salt composition. Our data indicate that the performance of various DNA dyes in qPCR is differently affected by salt composition of the PCR mixes. When genomic DNA fragment of mouse Thy-1 (864 bp) was amplified, SGI completely inhibited PCR in most of the PCR mixes. The only PCR mix which allowed efficient amplification of the Thy-1 fragment in the presence of SGI was mix IV, which differed from other mixes by the presence of both KCl and (NH_4_)_2_SO_4 _and the substitution of MgSO_4 _for MgCl_2_. This mix had pH 8.8, which also contributed to its performance; when pH of this mix was decreased to 8.0, SGI-mediated inhibition of PCR was noticed (data not shown). Besides pH, salt composition also plays a role because SGI completely inhibited amplification in PCR mix II, which has the same pH as mix IV. When other dyes were tested, amplification in mix IV was completely (EvaGreen) or partially (SYTO-9, SYTO-13, ResoLight) inhibited or remained unchanged (LCGreen and SYTO-82). In contrast to SGI, other dyes allowed amplification of the Thy-1 genomic fragment when present in PCR mix II. This mix is unique among others by containing 20 mM (NH_4_)_2_SO_4 _instead of KCl, enhanced concentration of Tris-HCl (75 mM) and inclusion of Tween 20 instead of Triton X-100 (Table 2). It should be mentioned that all PCRs were run under identical cycling conditions. Optimization of annealing temperature and cycling conditions for individual SGI-supplemented PCR mixes, however, resulted in no substantial improvement of PCR efficiency (data not shown). Neither was any improvement achieved by varying the concentration of Mg^2+^. Since the concentrations of deoxynucleotide triphosphates (dNTPs), DNA polymerase and anti-Taq monoclonal antibody (mAb) were identical in all PCR mixes tested, it is likely that an interaction of DNA dye with ionic environment is responsible for the observed differences in qPCR performance. Both divalent and monovalent cations bind to DNA and affect its physical properties [31]. Thus, ionic environment and DNA dyes could affect DNA denaturation, annealing of oligonucleotide primers to the DNA template and/or activity of Taq DNA polymerase, including its enhanced mutation frequency which could inhibit PCR [14]. Our data indicate, however, that error rate was not dramatically affected and therefore it is unlikely that it could contribute to the observed differences in PCR mixes of different salt composition. Neither was fidelity of the Taq DNA polymerase affected by the presence of various DNA dyes.

Under more stringent conditions, such as amplification of medium-size GC-rich genomic fragments from crude blood samples, no amplification was observed in various commercial and home-made PCR mixes with or without DNA dye, and no dramatic improvement was achieved with additives and/or procedures recommended for amplification of GC-rich templates. These included supplementing PCR mixes with DMSO, betaine, trehalose, 1,2-propanediol or 7-deaza-2'-deoxyguanosine 5'-triphosphate, performing hot-start PCR under "touchdown" conditions, or with recently introduced PCR enhancing coctail containing trehalose, L-carnitin and Nonidet P-40 (Figure 3). It is likely that PCR product formation under such conditions was compromised by the presence of inhibitors present in blood (e g. hemoglobin), concurrently with inadequate strand separation due to higher GC content and/or formation of secondary structures reflecting Hoogsteen base pairing between successive guanosine bases. Furthermore, DNA templates may create intramolecular stem loops formed during initial cycles of amplification, leading to formation of hairpin structures that are resistant to amplification by Taq DNA polymerase in subsequent cycles of PCR [32].

Interestingly, strong and specific amplification was observed when PCR mix II was supplemented with both 1 M 1,2-propanediol and 0.2 M trehalose. This mix allowed efficient amplification in the presence of all DNA dyes tested, including SGI. Trehalose has been previously shown to enhance the yield of the amplified PCR products, and it has been speculated that it acts through its thermostabilizing effect and/or lowering the template melting temperature [18,33]. However, it is unlikely that any of these two factors contributed individually to the observed effect of trehalose. In fact, the enzymatic activity of Taq DNA polymerase preincubated for 15 min at 95°C in mix II supplemented with 0.2 M trehalose (without blood inhibitors) was slightly decreased, rather than increased. Furthermore, although 0.2 M trehalose decreased melting temperature, this decrease was only marginal compared to the effect of other additives like DMSO or betaine which are routinely used as PCR enhancers.

1,2-propanediol at a final concentration 0.816 M has been recognized as an effective enhancer for amplification of medium-size GC-rich DNA sequences [24,34]. Although 1,2-propanediol surpassed 2.2 M betaine in its ability to amplify GC-rich templates [24] and decreased the melting temperature of dsDNA fragments (this study), it was unable on its own to surpass the inhibitory effect caused by the components present in the whole blood. Efficient amplification of such difficult-to-amplify templates was only achieved when 1,2-propanediol was combined with trehalose. It should be noted, that 1,2-propanediol slightly decreased enzymatic activity of Taq DNA polymerase. This inhibitory effect was however fully compensated by beneficial effects of 1,2-propanediol on PCR performance. Our finding of significant decrease of Taq DNA polymerase activity in samples supplemented with blood and even more with blood and 1,2-propanediol, and removal of this inhibitory effect after addition of trehalose suggest that trehalose acts by protecting the enzyme from negative interference of the blood inhibitors. Similarly, trehalose protected against the inhibitory effect of SGI, as indicated by enhanced amplification in the presence of both 1,2-propanediol and trehalose, compared to 1,2-propanediol alone.

In our previous study we found that SGI bound to ssDNA primers and interfered with annealing of the primers to DNA template; in this way SGI could, at least in part, contribute to its inhibitory effect on PCR [5]. In the present study we extended these tests to other DNA dyes and found that all of them bound to ssDNA oligonucleotides, as reflected by enhanced fluorescence. Importantly, for most of the dyes this binding was decreased by 1 M 1,2 propanediol. Extent of the inhibition depended on a combination of DNA dye used and GC content of the primers.

One of the PCR inhibitors present in blood is hemoglobin [17]. The inhibitory effect of hemoglobin on amplification of GC-rich DNA fragments was counteracted by addition of PT enhancer but not by 1,2-propanediol or trehalose alone. Although hemoglobin decreased melting temperature of dsDNA which was further decreased by the addition of 1,2-propanediol, it was still not sufficient for removal of its inhibitory effect. Interestingly, hemoglobin-containing samples supplemented with 1,2-propanediol or 1,2-propanediol and trehalose exhibited similar melting temperature, suggesting again that PT enhancer does not increase PCR performance solely by decreasing melting temperature.

Zhang and collaborators recently described a TCN coctail which in combination with inhibitor-resistant Taq DNA polymerase mutants enabled efficient amplification of high-GC content DNA targets directly from crude blood samples [27]. However, when wild-type Taq DNA polymerase was used for PCR with TCN, only a fraction of GC-rich DNA templates in whole blood was amplified. Under identical conditions, PCR mix supplemented with PT enhancer efficiently amplified all fragments analyzed in this study. These data indicate that PT is a superior enhancer for amplification of difficult templates when wild type Taq DNA polymerase is used. PT enhancer can be used as a universal enhancer suitable for normal targets, GC-rich targets (up to 79% GC) and/or targets in the presence of PCR inhibitors. PCR mix II supplemented with PT enhancer is suitable for DNA genotyping from whole blood without DNA extraction and is compatible with various DNA dyes. In order to get reasonable regression coefficients based on qPCR analyses of crude blood samples it is adviceable to dilute DNA templates present in human blood with e.g. mouse blood to keep the amount of the blood components in PCR constant. This will eliminate the problems with simultaneous dilution of the inhibitory (e.g. hemoglobin, immunoglobulins) and/or stimulatory (e.g. heparin) components which interfere with PCR performance, and/or recording of the fluorescence signal.

## Conclusions

This study shows that a combination of 1 M 1,2-propanediol and 0.2 M trehalose represents a unique enhancer which can be combined with various DNA dyes. The enhancer can be used for amplification of various DNA templates, including those which are GC-rich and present in crude specimens, where other enhancers such as DMSO or betaine fail.

## Methods

### Reagents, antibodies and plasmids

The origin and properties of DNA dyes are specified in Table 1. DMSO was obtained from Fluka Chemie Gmbh (Buchs, Switzerland). Taq DNA polymerase was produced as described [5] or obtained together with buffers of various composition from several manufacturers (Fermentas, Vilnius, Lithuania; Sigma-Aldrich, Prague, Czech Republic; Promega, Madison, USA). All other chemicals were from Sigma-Aldrich. The production of anti-Taq mAb which inhibits Taq DNA polymerase activity and is suitable for hot-start PCR has already been described [5]. The plasmid pMOL21 (4 kbp), carrying the *bla *gene for ampicillin resistance and r*ps*L gene, and *E. coli *K12 strain, MF101 [15], were obtained from Hisaji Maki (University of Tokyo, Japan).

### qPCR

Most of the experiments were conducted with Mastercycler ep realplex (Eppendorf, AG, Hamburg, Germany) according to the manufacturer's instructions. Reactions were performed in 20 or 25 μl volumes in twin.tec 96 real-time PCR plates (Eppendorf) sealed with heat sealing film (Eppendorf). Amplifications of cDNAs were performed in 5 μl reaction volumes in 384-well plates sealed with LightCycler 480 sealing foil (Roche Diagnostics) using LightCycler 480 (Roche Diagnostics, Mannheim, Germany). PCR mixes of different salt composition (Table 2) were prepared according to Taq DNA polymerase manufacturer's protocols and/or literature data [5]. Oligonucleotide primers, DNA template, DNA dyes and additives/enhancers were added immediately before the assay. For comparison, we also used iQ™ SYBR Green Supermix (Bio-Rad Laboratories, Hercules, CA, USA) and LC 480 SGI (Roche Diagnostics). DNA dyes were prepared from stock solutions as recommended by manufacturers and/or previous studies [2,3] and as summarized in Table 1. Primers used for amplification of genomic DNA fragments are indicated in Table 4. For cDNA amplification, the following primers were used (forward/reverse, [accession number; fragment size, GC content]): actin, 5'-GATCTGGCACCACACCTTCT-3'/5'-GGGGTGTTGAAGGTCTCAAA-3', [NM_007393.2; 138 bp, 58% GC]; GAPDH, 5'-AACTTTGGCATTGTGGAAGG-3'/5'-ATCCACAGTCTTCTGGGTGG-3', [XM_001473623.1; 69 bp, 52% GC]; ORMDL1_Fr.a, 5'-GGATCAGGGTAGAGCAAGG-3'/5'-AGCAGAGAAGCTGTGTTTAGG-3', [NM_145517.4; 171 bp, 43% GC]; ORMDL1_Fr.b, 5'-ACTCGTGTAATGAACAGCCG-3'/5'-GCCTTGCTCTACCCTGATCC-3', [NM_145517.4; 205 bp, 47% GC]. For amplification of 864 bp genomic DNA fragment of mouse Thy-1 gene, thermal cycling consisted of 94°C/1 min | 30 × [94°C/15 s | 56°C/15 s | 72°C/1 min]. For amplification of human or mouse genomic DNA fragments from crude blood samples, the cycling conditions were: 95°C/10 min | 40 × [94°C/15 s | 58°C/30 s | 72°C/1 min]. For cDNA amplification the cycling conditions were: 95°C/3 min | 40 × [95°C/10 s | 60°C/20 s | 72°C/20 s]. Melting curve analysis was carried out from 50°C to 95°C with 0.2°C increments. In some experiments, DNA amplicons were size-fractionated in 1% or 2% agarose gels stained with ethidium bromide (0.5 μg/ml) and evaluated as described [35]. Quantification cycle (Cq) values [36] were determined by automated threshold analysis. PCR efficiencies (*E*) were determined from dilutions of DNA and calculated from the slopes of the standard curves according to the equation *E *= 10^-1/*a *^- 1, where *a *is the slope of the corresponding standard curve.

### Genomic DNA

Mouse genomic DNA was isolated from tails of C57BL/6J mice as previously described [37]. As a source of human genomic DNA, whole human blood was collected into heparin (20 U/ml), 0.38% sodium citrate or 2.7 mM EDTA, and stored in small aliquots at -70°C. All these experiments were approved by the ethical committee of the Institute of Molecular Genetics. The mass of the haploid mouse and human genome (C-value) is ~3.3 pg and ~3.5 pg, respectively [38]; this indicates that 1 ng of mouse or human genomic DNA contains approximately 303 and 286 copies of a single-copy gene. These numbers were used for generation of standard curve of Cq values from amplification plots versus log copy number.

### RNA extraction and cDNA synthesis and analysis

RNA was extracted from mouse bone marrow-derived mast cells cultured under standard conditions [39] using RNeasy Mini Kit (Qiagen, Hilden, Germany). The amount of RNA was determined by spectrophotometer ND-100 (NanoDrop Technologies, Wilmington, DE). Single-stranded cDNA was synthesized by means of mouse moloney leukemia virus reverse transcriptase (Invitrogen, Carlsbad, CA, USA) according to manufacturer's instructions using 10 μg of isolated RNA and 50 ng of random hexamers per reaction.

### Fluorescence measurements and melting temperature determination

PCR mix II without Taq DNA polymerase, dNTPs and anti-Taq antibody was supplemented with various additives/enhancers, DNA dyes and ssDNA oligonucleotide PCR primer TNF, 5'- TAAAACGACGGCCAGTGAATTC-3' (45.5% GC) or primer No 7, reverse (Table 4; 72.2% GC). The mixtures (25 μl) were transferred into white wells of the 96-well PCR plate, heat-sealed, and fluorescence reading was carried out on Mastercycler ep realplex (SGI filter set). The samples were heated at 95°C for 2 min, then cooled to 50°C and subjected to fluorescence reading. For determination of melting temperatures, the samples were prepared as above except that dsDNA was formed by adding oligonucleotide mixture of TNF and anti-TNF (5'-GAATTCACTGGCCGTCGTTTTA-3') or primer No 7, reverse (Table 4) and anti-primer No 7, reverse (5'-AGACCCGCCTCACGAGCC-3'). In some experiments human hemoglobin (Sigma-Aldrich) at a final concentration 37.5 μM was also added. The samples were heated at 95°C for 2 min and then cooled to 30°C. Temperature-dependent changes in fluorescence obtained during heating from 30°C to 95°C (0.2°C increments) were determined by Mastercycler ep realplex. Melting temperatures were determined from melting curves.

### DNA polymerase fidelity assay

The fidelity assay was based on streptomycin resistance of *rps*L mutants [15]. Standard PCRs (50 μl) containing mixes of different composition, as indicated in Table 2, were supplemented with 0.2 μM of each primer and 1 ng of template DNA (pMOL21 plasmid linearized with ScaI). In some experiments various DNA dyes at concentrations specified in Table 1 were also added. The PCR with KOD hot start polymerase was performed according to the manufacturer's instruction (Novagen, Darmstadt, Germany). The following primers were used: biotin-5'-AAAAACGCGTCACCAGTCACAGAAAAGCATCTTAC-3' (forward sequence) and 5'-AAAAACGCGTCAACCAAGTCATTCTGAGAATAGT-3' (reverse sequence) [40]; MluI restriction sites are underlined. The standard PCR conditions were: 94°C for 2 min, followed by 25 cycles at 94°C for 15 s, 58°C for 30 s and 68°C for 5 min. The concentration of amplified DNA was determined by means of Quant-iT dsDNA HS Assay Kit (Invitrogen), and the number of template doublings was estimated. The PCR products were collected using streptavidin magnetic beads (Dynabeads M-280 Streptavidin, Invitrogen). Briefly, 100 μl aliquots of the beads were rinsed with washing solution (5 mM Tris-HCl, pH 7.5, 0.5 mM EDTA, 1 M NaCl), resuspended in 180 μl washing solution, combined with 20 μl of the PCR amplified DNA, and incubated under gentle rotation at room temperature. After 30 min, the beads were washed, resuspended in 100 μl of the corresponding enzyme mix and treated with 10 units of MluI at 37°C with gentle rotation overnight. The beads were collected using magnetic stand, and the supernatant was fractionated by electrophoresis in 0.8% agarose gel. The DNA fragment was isolated using TaKaRa Recochip (TaKaRa Biomedicals, Kyoto, Japan), precipitated in ethanol, lyophilized and dissolved in 20 μl of sterile water purified with Milli-Q Advantage A10 (Millipore, Molsheim, France). The purified DNA was self-ligated with T4 DNA ligase and transformed into MF101 competent cells. Half of the transformants were plated with ampicillin (100 μg/ml) to determine the total number of transformed cells; the remaining half on plates with ampicillin and streptomycin (100 μg/ml each) to determine the total number of *rps*L mutants. The mutation frequency was determined by dividing the total number of mutants by the total number of transformed cells. The error rate was calculated by dividing the mutation frequency by 130 (the number of amino acids that cause phenotypic changes in *rps*L), and the number of template doublings [15].

### DNA polymerase activity

Activity of Taq DNA polymerase was assayed by measuring the conversion of radiolabeled dATP into acid insoluble DNA as previously described [41] with some modifications. Activated salmon testes DNA (Sigma-Aldrich) was prepared by exposing the DNA to low concentrations of pancreatic DNase [42]. Reaction mixture (100 μl) contained DNA polymerase 50 U/ml, 75 mM Tris-HCl, pH 8.8, 20 mM (NH_4_)_2_SO_4_, 0.01% Tween 20, 2.5 mM MgCl_2_, 100 μM dATP, 200 μM dGTP, 200 μM dCTP, 200 μM dTTP, activated salmon testes DNA (0.2 mg/ml), and various additives [10% (final concentration) human sodium citrate-treated blood, 0.2 M trehalose and/or 1 M 1,2-propanediol]. The samples were denatured for 15 min at 95°C then cooled to 72°C and supplemented with [α-^32^P]dATP (14.8 kBq; 111TBq/mmol; MP Biomedicals, Irvine, CA). The mixtures were incubated for 30 min at 72°C and the reactions were then stopped by the addition of 100 μl of stop solution (150 mM sodium pyrophosphate and 100 mM EDTA, pH 8.0). DNA was precipitated by the addition of 150 μl of ice-cold 25% trichloracetic acid. After 15 min on ice, the samples were vacuum filtered on type A/E glass fiber filters (25 mm; Pall Corporation, Ann Arbor, MI) pre-wet with stop solution. Precipitated DNA retained on the disc was washed with 5 ml ice-cold 10% trichloracetic acid followed by 10 ml of ice-cold 96% ethanol. The filters were air dried and the radioactivity was measured in 10 ml scintillation fluid BetaMax ES (MP Biomedicals) in a scintillation counter with QuantaSmart software (Perkin Elmer, Waltham, MA).

### Statistical analysis

Statistical analysis of intergroup differences was performed using Student's t-test.

## List of abbreviations

qPCR: quantitative real-time PCR; SGI: SYBR green I; PT enhancer: 1 M 1,2-propanediol and 0.2 M trehalose; ds: double-stranded; ss: single-stranded; bp: base pairs; LC 480 SGI: LightCycler 480 SYBR Green I Master; DMSO: dimethyl sulfoxide ; TCN: 0.3 M D-(+)-trehalose, 0.24 M L-carnitine, and 0.4% Nonidet P-40; TNF: tumor necrosis factor; dNTP: deoxynucleotide triphosphate; mAb: monoclonal antibody; Cq: quantification cycle; E: efficiency.

## Competing interests

The authors declare that they have no competing interests.

## Authors' contributions

HH carried out experiments with PT enhancers. IP performed DNA polymerase fidelity assays and wrote the corresponding part of the manuscript. GMS carried out the experiments presented in Figure 1. JE performed the experiments presented in Figure 5. VB conceived the experiments with cDNA analysis and wrote the corresponding part. LD and PD conceived the study and wrote the manuscript. All authors read and approved the final manuscript.
